# Morphology of the Vasculature and Blood Supply of the Brown Adipose Tissue Examined in an Animal Model by Micro-CT

**DOI:** 10.1155/2020/7502578

**Published:** 2020-02-27

**Authors:** J. Mrzilkova, P. Michenka, M. Seremeta, J. Kremen, J. Dudak, J. Zemlicka, V. Musil, B. Minnich, P. Zach

**Affiliations:** ^1^Specialized Laboratory of Experimental Imaging of the Third Faculty of Medicine, Charles University and the Institute of Experimental and Applied Physics and Faculty of Biomedical Engineering, Czech Technical University in Prague, Prague, Czech Republic; ^2^Department of Anatomy, Third Faculty of Medicine, Charles University, Prague, Czech Republic; ^3^Institute of Experimental and Applied Physics, Czech Technical University in Prague, Prague, Czech Republic; ^4^Faculty of Biomedical Engineering, Czech Technical University in Prague, Kladno, Czech Republic; ^5^Centre of Scientific Information, Third Faculty of Medicine, Charles University, Prague, Czech Republic; ^6^Department of Biosciences, Vascular & Exercise Biology Unit, University of Salzburg, Austria

## Abstract

We performed micro-CT imaging of the vascular blood supply in the interscapular area of the brown adipose tissue in three mice with the use of intravascular contrast agent Aurovist™. Resulting 3D data rendering was then adapted into 2D resolution with visualization using false color system and grayscale images. These were then studied for the automatic quantification of the blood vessel density within this area. We found the highest most occurring density within arterioles or venules representing smaller blood vessels whereas with the increase of the vessel diameters a lower percentage rate of their presence was observed in the sample. Our study shows that micro-CT scanning in combination with Aurovist™ contrast is suitable for anatomical studies of interscapular area of brown adipose tissue blood vessel supply.

## 1. Introduction

In mice and other rodents, brown adipose tissue (BAT) is mainly located in the interscapular, axial, and perirenal regions and remains activated during adulthood [1]. Its main characteristics are a large number of mitochondria and a multilocular storage of fat. In rodents, activation or expansion of BAT is associated with protection from obesity and increased insulin sensitivity.

Besides BAT, rodents also possess many depots of white adipose tissue (WAT). The actual function of WAT is to store energy in the form of triglycerides to provide the body with energy when needed [2]. Both WAT and BAT are highly vascularized tissues. The adipose vasculature fulfills a number of important functions in adipose depots, including the supply of oxygen and nutrients. Apart from that, the adipose vasculature also plays an important role in the removal of metabolic waste products and CO_2_. These features are of particular significance in the thermogenically highly active BAT. In all locations under inspection, interscapular BAT depot is of main significance as it allows drainage of warmed blood via Sulzer's vein (4^th^ thoracic vein) to the heart [3], which allows optimal redistribution of heat to the other parts of the body [4]. More cranially located interscapular BAT (I-BAT) also possesses venous return into the inner vertebral sinus between levels T2-T6 of the spinal segment in the rat [5]. BAT thermogenesis function also plays an important role in the regulation of the body homeostasis in a temperature hostile environment.

A previous study [5] examined the vascularization of the I-BAT by application of latex and polyvinyl resins into the blood vessels of the rat.

In fact, in order for BAT to expand, angiogenesis is an important prerequisite. Similarly, the browning of WAT upon cold exposure is also associated with angiogenesis and an increase in vascular density [2].

Metabolically, less active WAT shows a lower vessel density compared to metabolically active BAT expressing high vessel density, energy expenditure, and heating [6].

The positive feedback drug delivery system may cause the WAT browning process. Released rosiglitazone and prostaglandin E2 analog promoted transformation of WAT into brown-like adipose tissue and stimulate angiogenesis. This facilitates the homing of targeted nanoparticles to adipose angiogenic vessels, thereby amplifying their delivery and hence expediting the WAT browning process [7].

## 2. Materials and Methods

### 2.1. Tissue Sample Origin

For our experiment, 3 specimens of C57BL/6 type mice were used instead of rats so that the animals fit inside the micro-CT holder tube. After 24 hrs of food denial, mice were anesthetized with isoflurane gas injected through the retrobulbar sinus with Aurovist™ nanogold particles (15 nm), an intravascular contrast agent. After euthanization, the whole bodies of the 3 mice were fixated in a row of ascending ethanol concentrations (50% ethanol for 24 hrs, 75% for 24 hrs, and 98% for 168 hrs) and scanned in the micro-CT.

### 2.2. Micro-CT Apparatus

The presented data have been acquired using a custom adapted MARS micro-CT scanner. The parameters of the used system were described in details in our previous publications [8–10]. The system is equipped with a Kevex™ PXS-11 X-ray tube and Timepix detector in Quad configuration (four read-out chips with a common 300 *μ*m thick silicon sensor providing a sensitive area of 28 × 28 mm) housed in a rotating gantry. The system provides spatial resolution within the range of approx. 28-45 *μ*m depending on the actual sample dimensions.

### 2.3. Imaging Protocol

Each scan consisted of 720 equidistant X-ray projections with 0.5-degree angular step and resolution of 44 *μ*m. The source was operated at 70 kVp (150 *μ*A), and a 140 *μ*m thick aluminum beam filter was used.

The acquired data were processed by a beam hardening correction for photon-counting detectors prior to the CT reconstruction [11]. The CT reconstruction was performed using the NRecon software [12]. The obtained micro-CT slices were then visualized in CTVox software (version 3.3.0 r1401, 64 bit version, Bruker) [13] and Dragonfly software (software version 4.1.0.647) [14], and the quantitative analyses of the BAT vasculature were carried out using the CT-Analyzer (version 1.17.7.2+, 64 bit version, Bruker).

### 2.4. Micro-CT Image Post Processing

An interscapular area with its BAT and vasculature was selected as the ROI, without bony structures (scapula, vertebral column, and skull). The position of the BAT and vasculature in the interscapular area was allocated according to Cinti [15]. The ROI (I-BAT) was manually delineated by an experienced anatomist in the software program Dragonfly. The exported ROI from Dragonfly software was then analysed in the CT-Analyzer by 3D analysis command. The percentages of vessels diameters, e.g., vascular thickness (including arteries and veins), were acquired in this ROI (Table 1).

### 2.5. Ethical Statement

The study was approved by the local Ethical Committees of the First and Third Faculty of Medicine, Charles University, Prague, Czech Republic (24185/2019). The animals were treated in accordance with the guidelines defined by the ethical committee, which follows the National Advisory Committee for Laboratory Animal Research (NAC LAR) guidelines. Handling of animals was done in accordance with the Helsinki Declaration (seventh revision, 2013).

## 3. Results

**Figure 1:**
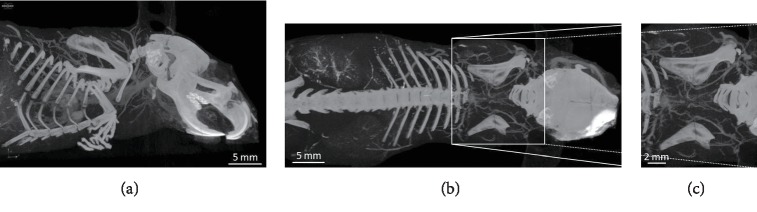
Greyscale micro-CT scans of mouse I-BAT vasculature. Lateral view (a), overall posterior view (b), and detailed view of interscapular area (c). Scans are done in NRecon software and CTVox visualizer.

**Figure 2:**
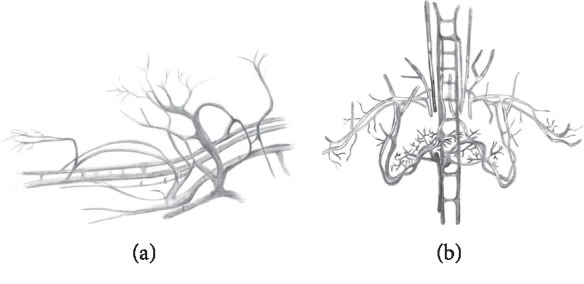
Lateral view (a) and posterior view (b) of the I-BAT vascularization in latex-injected animals (adapted by C. Sudova from Smith and Roberts [5]).

**Figure 3:**
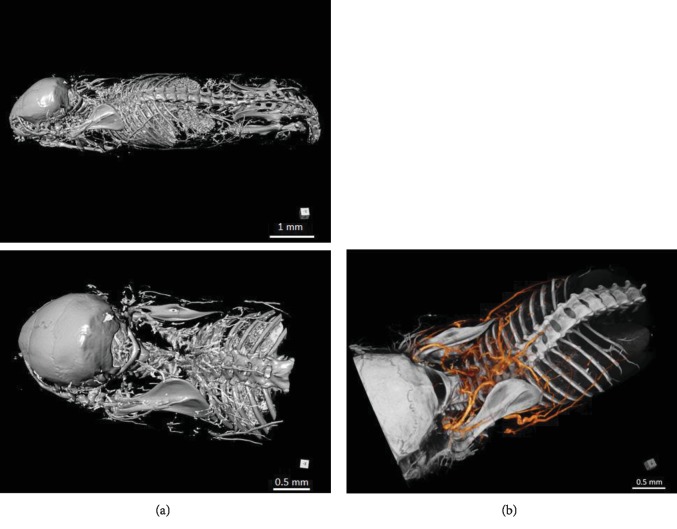
Micro-CT scans of mouse I-BAT vasculature. (a) Lateral view and (b) posterolateral view of interscapular region with colored vessels. Images were generated using Dragonfly software [12].

We did micro-CT scanning of a post mortem mouse with ethanol fixation and Aurovist™ intravascular contrast. 2D micro-CT images of the I-BAT vasculature in the lateral and posterior views (Figure 1) were compared to similar vasculature drawings, adapted according to Smith and Roberts [5] (Figure 2). For post processing of I-BAT grayscale images and in-depth visualization including the surface of the I-BAT vasculature from a whole mouse, Dragonfly software was used (Figure 3). The best visible structures found were a mesh of a small capillary network in the interscapular area, located dorsally from the vertebral column. These small blood vessels became even more visible in the ROI since the contrast of the vertebral column did not interfere with the Aurovist™ contrast. The vasculature of the I-BAT was also captured in the form of 3D ROI (see supplementary data ([Supplementary-material supplementary-material-1]): 3D projection of the I-BAT ROI vasculature in rotation movie in all three planes: https://drive.google.com/file/d/1tfFZQRj0AxhlHsMELpOOUuXXkrJIUQcQ/view).

**Figure 4:**
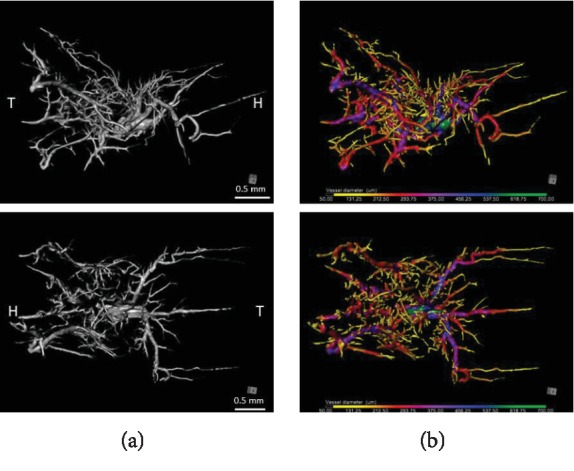
(a) Black and white volume rendering of the segmented BAT structures. Lateral view of the right side (top) and above view (below) of the ROI (I-BAT vasculature). Only blood vessels are depicted. In the core, the microvasculature of the I-BAT is located while on the periphery there are larger vessels serving the blood supply. (b) Identical picture to the left side, in colors, representing all vessel diameters. Each color corresponds to specific vessel diameter (in micrometer scale below the picture). Images were generated using Dragonfly software [12]. H: head; T: tail.

2D images of the interscapular area of I-BAT only (Figure 4) were then adapted from 3D ROI data (only vascular tissue).

The thickness of vessels in I-BAT was analysed in one representative animal (with the best detailed I-BAT visualization). We found the highest percentage distribution (24.84% and 20.79%) for smaller and below average size blood vessel diameters (134-223 *μ*m and 312-401 *μ*m), followed by distribution (18.73% and 16.05%) for blood vessel diameters (223-312 *μ*m and 45-134 *μ*m—smallest blood vessel diameter), and the lowest percentage distribution (4.18% and 3.3%) for the highest vessel diameters (490-579 *μ*m and 579-668 *μ*m) (Table 1).

## 4. Discussion

We studied the structure of the vasculature of the I-BAT in mice by micro-CT imaging using the Aurovist™ contrast agent. A similar description of the anatomy of the vasculature of the I-BAT in latex-injected animals was part of a larger study focusing on thermoregulation in the BAT [5]. Recent studies on the vasculature of the I-BAT mostly used light microscopy, MRI, or PET imaging methods. In this context, I-BAT vascular density was examined by microangiography during norepinephrine infusion prior to the casting of Microfil® in the aorta demonstrating the lipophilic xenon gas effect on BAT [16]. Besides increased perfusion of the tissue compared to controls, images showed massive collaterals between WAT and BAT. Light microscopy was then used to assess differences in vascular density in treated and control mice after the excised tissue had been optically cleared. In the past, several different methods of BAT imaging were used to quantify its lipid content, to show metabolic activity in defined situations or for pure imaging purposes. For example, an in vivo quantitative lipidic map of brown adipose tissue by chemical shift imaging at 4.7 tesla was documented by Lunati et al. [17]. The main advantages of MRI are the following: (i) it is a noninvasive method, allowing to image the spatial distribution of the lipid, and (ii) in most cases, MRI can be used repeatedly on the same animal enabling to observe changes of fat tissues in different metabolic or aging conditions and thus to carry out longitudinal studies maintaining the same laboratory animals [18]. The older MRI 2D spin echo method used by Lunati et al. [17] necessitates a high magnetic field in order to gain proper spin echo performance and was composed of time-consuming (20 minutes) acquisitions of two separate fat-only and water-only chemical-shift-selective images. These inconveniences initiated the development of new methods of MRI imaging. Hu et al. [19] used IDEAL-MRI; however, other limitations by means of isotropic resolution of 0.6 mm, false signals, especially in small areas of contact of fat tissue and low fat tissue and others appeared. Micro-PET as compared to other imaging methods, described by Marzola et al. [20] or DiFilippo et al. [21], is mostly affected by limited spatial resolution (1 mm), expensive radiotracers, and the need for a controlled environment.

Software CT-Analyzer analyses make it possible to automatically calculate trabecular thickness in the ROI. We used this program to calculate differences between percentage distributions of blood vessels by their diameter in I-BAT. We found the highest percentage volume of vessels with diameters in the interval between 134 and 401 *μ*m. This was valid for the I-BAT because of its high metabolic activity depending on the microvascular blood supply. This implies that the system of blood vessels with average and smaller diameters is the most important for the direct vascularization of I-BAT (Table 1). These results were obtained after ethanol fixation of the specimens prior to micro-CT scanning. The reason for this might be that ethanol fixation caused dissolution of the fat pad located in the interscapular area in the vicinity of I-BAT vessels. This way reached an increase in the effect of the Aurovist™ contrast agent in the vessels not surrounded by the adipose tissue. Therefore, we suggest that post mortem visualization of the I-BAT vessels gives better results compared to in vivo methods where native adipose tissue blocks the vessels' resolution (unpublished results). Vascular thickness or density is often studied with regard to the angiogenesis of WAT or BAT [22] or in metabolism studies [23]. Such studies rarely include anatomical details about vascular diameters; rather, they provide only information about vascular density [6].

The advantages of micro-CT scanning with Aurovist™ are more precise and detailed tissue samplings which in turn could serve for further histological and immunohistochemical studies of I-BAT blood supply [24]. When enlarging the ROIs of the scans, specifically, I-BAT subregions with the most dense microcirculation (the most metabolically active part of the I-BAT) can be targeted which enables (i) local perfusion of the tissue, (ii) changes in its thermodynamics, and (iii) levels of various chemical substances in the intra- or extravascular space. The limitation of our study was the spatial resolution achieved with the used micro-CT scanner (44 *μ*m). For that reason, we could not measure the vascular thickness of capillaries (usually below 10 *μ*m, 10-20 *μ*m, and above 20 *μ*m) but blood vessels with larger diameters, classified as arterioles or venules. The presented pilot measurements demonstrate the feasibility of BAT visualization using micro-CT techniques. The detail detectability can be significantly improved using a different type of a micro-CT scanner. The current state-of-the-art micro-CT technology allows achieving spatial resolution better than 10 micrometers, especially in the case of a post mortem scan.

Our study shows the possibility to visually navigate through the tissue prior to histological sampling, microinjection of contrast matter, or measurement of metabolic activity in the area. Specifically, it would be suitable for visual navigation in experiments dealing with vessel occlusion in I-BAT and its metabolic activity.

## 5. Conclusion

We clearly showed that the Aurovist™ contrast agent is appropriate for the use in I-BAT vasculature studies with micro-CT and also that the resolution of blood vessels is high enough for automated vascular thickness measurements. We found that vascular thickness (diameter) at the level of arterioles or venules types of blood vessels was highest in I-BAT in the interval 134-401 *μ*m and lowest in the interval 490-668 *μ*m (highest diameter of measured vessels). In human studies on vascular changes which occur in some metabolic diseases, it is possible to use Aurovist™ contrast in micro-CT but only after the surgical removal of the pathologically changed tissue, e.g., fingers from a diabetic foot.

## Figures and Tables

**Table 1:** Vascular thickness (refers to the inner diameter of the blood vessels without its walls) distribution in interscapular BAT in one mouse example. Values are split into 7 intervals within 4 categories: (i) *vascular thickness* shows vessel diameters in 7 intervals, (ii) *average vascular thickness* shows average diameter of the vessels, (iii) *volume of vessels* shows the total volume of vessels within given interval, and (iv) *percentage volume of vessels* shows numbers of vessels in intervals out of 100% of all measured vessels.

Vascular thickness (*μ*m)	Average vascular thickness (*μ*m)	Volume of vessels (mm^3^)	Percentage volume of vessels (%)
44.561 ≤ 133.68	89.12	2.82	16.05
133.69 ≤ 222.81	178.25	4.37	24.84
222.82 ≤ 311.93	267.37	3.29	18.73
311.94 ≤ 401.05	356.49	3.66	20.79
401.06 ≤ 490.17	445.61	2.13	12.07
490.18 ≤ 579.30	534.74	0.736	4.18
579.31 ≤ 668.42	623.86	0.587	3.33

## Data Availability

The data used (one hand drawing, three micro CT images and one Table) to support the findings of this study are included within the article. There is also http link in the text of article with the 3D rotatory images, located on the Google Drive.

## Abbreviations

BAT: Brown adipose tissue
I-BAT: Interscapular brown adipose tissue
WAT: White adipose tissue
ROI: Region of interest
RGB: Red-green-blue
IDEAL-MRI: Iterative decomposition with echo asymmetry and least squares estimation of MRI
PET: Positron emission tomography.

## Supplementary Materials

Supplementary Materials 3D projection of the I-BAT ROI vasculature in rotation movie in all three planes.
