# Supplementary material for: Morphology of the Vasculature and Blood Supply of the Brown Adipose Tissue Examined in an Animal Model by Micro-CT
Source: Biomed Res Int. 2020 Feb 27;2020:7502578. doi: 10.1155/2020/7502578 (PMC7064829; doi:10.1155/2020/7502578)
Supplement: Supplementary Materials — 3D projection of the I-BAT ROI vasculature in rotation movie in all three planes. [file 7502578.f1.docx]

https://drive.google.com/file/d/1tfFZQRj0AxhlHsMELpOOUuXXkrJIUQcQ/view
